# Role of the GalNAc-galectin pathway in the healing of premature rupture of membranes

**DOI:** 10.1186/s10020-024-00908-6

**Published:** 2024-09-04

**Authors:** Jia-Le Chen, Lou Liu, Xin-Rui Peng, Yan Wang, Xiang Xiang, Yu Chen, De-Xiang Xu, Dao-Zhen Chen

**Affiliations:** 1 The School of Public Health, Anhui Medical University, Hefei, China; 2 Wuxi Maternity and Child Health Care Hospital, Wuxi, China; 3 Department of Laboratory, Haidong No.2 People’s Hospital, Haidong, China; 4 Hospital Infection Management Section, Changzhou Wujin Hospital of Traditional Chinese Medicine, Changzhou, China; 5 Department of obstetrics, Longgang District Maternity & Child Healthcare Hospital of Shenzhen City, Shenzhen, China

**Keywords:** PROM, N-acetyl-d-galactosamine, Galectin, Healing

## Abstract

**Background:**

Premature rupture of the membranes (PROM) is a key cause of preterm birth and represents a major cause of neonatal mortality and morbidity. Natural products N-acetyl-d-galactosamine (GalNAc), which are basic building blocks of important polysaccharides in biological cells or tissues, such as chitin, glycoproteins, and glycolipids, may improve possible effects of wound healing.

**Methods:**

An in vitro inflammation and oxidative stress model was constructed using tumor necrosis-α (TNF-α) and lipopolysaccharide (LPS) action on WISH cells. Human amniotic epithelial cells (hAECs) were primarily cultured by digestion to construct a wound model. The effects of GalNAc on anti-inflammatory and anti-oxidative stress, migration and proliferation, epithelial-mesenchymal transition (EMT), glycosaminoglycan (GAG)/hyaluronic acid (HA) production, and protein kinase B (Akt) pathway in hAECs and WISH cells were analyzed using the DCFH-DA fluorescent probe, ELISA, CCK-8, scratch, transwell migration, and western blot to determine the mechanism by which GalNAc promotes amniotic wound healing.

**Results:**

GalNAc decreased IL-6 expression in TNF-α-stimulated WISH cells and ROS expression in LPS-stimulated WISH cells (*P* < 0.05). GalNAc promoted the expression of Gal-1 and Gal-3 with anti-inflammatory and anti-oxidative stress effects. GalNAc promoted the migration of hAECs (50% vs. 80%) and WISH cells through the Akt signaling pathway, EMT reached the point of promoting fetal membrane healing, and GalNAc did not affect the activity of hAECs and WISH cells (*P* > 0.05). GalNAc upregulated the expression of sGAG in WISH cells (*P* < 0.05) but did not affect HA levels (*P* > 0.05).

**Conclusions:**

GalNAc might be a potential target for the prevention and treatment of PROM through the galectin pathway, including (i) inflammation; (ii) epithelial-mesenchymal transition; (iii) proliferation and migration; and (iv) regression, remodeling, and healing.

## Introduction

Premature rupture of membranes (PROM) is a critical perinatal complications often leading to preterm birth(Tchirikov et al. 2018). Clinically, the management of PROM involves a dichotomy of approaches: the termination of pregnancy or the expectant management in hopes of spontaneous membrane resealing, a phenomenon observed infrequently(Ronzoni et al. 2022). In contrast, amniotic fluid leaks following amniocentesis typically exhibit a self-limiting nature, often resolving without intervention, highlighting a distinct disparity in the natural history of these conditions(Johnson et al. 1990). Hence, the prevailing clinical strategy for managing PROM, particularly before 32 weeks’ gestation, is expectant management.

The fetal membranes consist of three juxtaposed layers: the amniotic and chorionic membranes of fetal origin and the meconium of maternal origin (Parry and Strauss 1998), of which the amniotic layer is the predominant load-bearing structure. Unlike wound repair (Overmiller et al. 2022), the amniotic membrane contains no blood vessels or nerve endings (Pasquier and Doret 2008), and the amniotic wound healing process is not accompanied by vascular injury or granulation formation (Mogami and Word 2020; Sonnemann and Bement 2011). Thus, amniotic membrane healing consists of only four overlapping processes: (i) inflammation; (ii) epithelial-mesenchymal transition; (iii) proliferation and migration; and (iv) regression, remodeling, and healing.

Currently, the clinical management aims to improve neonatal prognosis by prolonging the time between rupture and delivery (Quintero et al. 1996). However, only a small percentage (7.7-9.7%) of pregnant women with premature rupture of membranes will heal spontaneously(Mogami and Word 2020). Research into amniotic membrane regeneration is expected to shed light on potential treatment strategies for PROM. The use of sugar to promote wound healing is one of the earliest known methods (Forrest 1982). In 1679, Scultetus used icing sugar to clean wounds (Pieper and Caliri 2003). In 1714, Zoinin promoted the value of sugar in promoting the healing of wounds and ulcers (Dawson 1996). In 1980, Herszage and colleagues described the use of simple granulated sugar in the treatment of infected wounds and epidermal injuries with a healing rate of 99.2% in 120 patients. In conclude, the use of sugars in promoting wound healing has been well-documented, dating back to the 17th century. Glucose, galactose, glucosamine, and galactosamine have been shown to foster wound tissue healing. Recent metabolomics analyses have implicated these sugars in the context of PROM, particularly noting the diminished expression of GalNAc—a monosaccharide—in individuals with PROM. Despite these findings, the precise function of GalNAc about PROM remains elusive. GAG saccharides, such as glucose, galactose, glucosamine, and galactosamine have been shown to promote wound tissue healing (Migone et al. 2022).

Our previous study showed by untargeted metabolomics analysis that multiple metabolic pathways are involved in vaginal microecological dysregulation that may lead to PROM, and GalNAc, a representative metabolite of monosaccharides, was expressed at lower levels in the PROM group Unfortunately, very little is known about the role of GalNAc in PROM. (Liu et al. 2021a, b). We hypothesised that GalNAc may play an important role in amniotic tissue healing to prevent the development of premature rupture of membranes.

## Methods

### IRB approval

The study was approved by the Ethics Committee of Wuxi Maternal and Child Health Hospital and registered in the China Clinical Trials Registry (registration number: chictr2000034721).

### Human amniotic epithelial cell culture

Human amniotic membranes were obtained from the fetal membranes of women who delivered by cesarean section with family consent and who were HIV, hepatitis A, hepatitis B, and syphilis negative and free of PROM. The amniotic layer was stripped from the chorionic villus layer and digested with 0.2% collagenase and 0.25% trypsin. The dispersed cells were inoculated in the medium at a density of 3–5 ×10^6^ cells per T75 and incubated at 37 °C, 5% CO_2_ until 80–90% confluence. The hAECs were passaged and the 3rd-5th generation of cells were used in this study.

### Measurement of cell viability

The hAECs and WISH cells were inoculated in 6-well plates and incubated overnight at 37 °C, 5% CO_2_. GalNAc was added to each well and normal incubation was continued for 24 h. CCK-8 solution was added to each well, and a blank control was established. After incubation for 1 h in a constant temperature incubator, cell viability was determined by measuring OD at 450 nm with an enzyme marker by plotting the proliferation curve.

### ROS detection

WISH cells were inoculated in 6-well plates and incubated overnight at 37 °C, 5% CO_2_. Cells were treated with 1 µg/ml LPS for 2 h and then incubated with GalNAc for 2 h. PBS washing was repeated 3 times and finally medium containing 10 µM DCFH-DA was added. Cells were incubated at 37 °C for 20 min, followed by washing with PBS to remove the free probe, and finally photographed for observation using an inverted fluorescence microscope.

### ELISA

WISH cells were inoculated in 6-well plates and incubated overnight at 37 °C, 5% CO_2_. Cells were treated with 10 ng/ml TNF-α for 2 h and then incubated with GalNAc for 2 h. The supernatant of cells was aspirated and centrifuged at 4 °C, 16,000 g for 20 min, and finally removed. The total protein content of samples was determined by BCA assay using an ELISA kit.

### Immunofluorescence

WISH cells were inoculated at approximately 80% confluence in 24-well plates and incubated overnight at 37 °C, 5% CO_2_. Cells were treated with GalNAc for 2, 4, 8, and 24 h. Cells were fixed in 4% paraformaldehyde for 10 min. After washing with PBS, cells were incubated with vimentin primary antibody (Abcam) for 2 h at room temperature and with secondary antibody (Abcam) and fixed with fixative plus DAPI dye. Images were acquired by inverted fluorescence microscopy and generated by Image J software.

### Scratch test

3–5 generations of hAECs were taken, cells were suspended in serum-free medium, and concentration was adjusted to 5 × 10^5^ /ml for inoculation in 6-well plates. At the confluence, the center of each well was scratched with a 10 µl tip (epithelial cells). The width of the wound was measured under the microscope at 3 points per field of view, and finally processed by Image J software and averaged.

### Transwell experiment

WISH cells at the logarithmic growth stage were suspended in a serum-free medium and the concentration was adjusted to 2.5 × 10^5^ /ml. 800 µL of medium containing 10% serum was added to the lower chamber and 100 µL of cell suspension was added to the upper chamber and incubated for 24 h. The lower surface was immersed in poly methanol solution, fixed for 30–60 min, and stained with crystal violet under an orthomosaic microscope. The cell migration was observed and photographs were taken at 3 random fields of view per well. Finally, the number of cells on the lower surface of the PET membrane was counted and averaged.

### Western blot

The hAECs and WISH cells were inoculated in 6-well plates and incubated overnight at 37 °C, 5% CO_2_. GalNAc was added and incubation was continued for 24 h. The solution was aspirated and washed 3 times with PBS. 30 µL of cell lysate containing PMSF was added and cells were collected on ice. It was then transferred to EP tubes and shaking lysis on ice was continued for 30 min. The lysate was centrifuged at 4 °C 16,000 g for 20 min and the supernatant was extracted by the BCA method to determine the total protein content of samples. The relative protein content was determined by the western blot method.

### DMMB

WISH cells were inoculated in 6-well plates and incubated overnight at 37 °C, 5% CO_2_. GalNAc was added and incubation was continued for 24 h. Cells were collected on ice, added with 1 ml of PBS, mixed, transferred to a pre-chilled 1.5 ml EP tube, and centrifuged at 4 °C 300 g for 5 min, and the supernatant was discarded. It was then added with 1 ml of 2% papain, followed by a vortex shake vigorously for 1 min, incubated for 16 h at 56 °C in a constant temperature water bath, incubated for 10 min at 90 °C in a constant temperature water bath, and centrifuged for 10 min at 4 °C 16,000 g, and the supernatant was removed. Besides, it was added with 1 ml of DMMB staining solution, followed by vortex shake for 15 s, incubated for 30 min at room temperature and avoiding light, followed by vortex shake for 15 s every 5 min. It was immediately centrifuged for 10 min at 16,000 g, and the supernatant was removed. It was then added with 1 ml of propanol, followed by vortex shake for 15 s, incubated for 5 min at room temperature and avoid light, and transferred to an enzyme plate to obtain the OD value with a full wavelength enzyme marker (656 nm). The curve was plotted to determine the concentration of cytosolic glycosaminoglycan.

### Statistics

Statistical analysis of normally distributed data was performed using ANOVA with Tukey’s multiple comparison test and t-test. Statistical values were calculated using PRISM. *P* values less than 0.05 were considered significant. Data are expressed as mean ± SEM.

## Results

### Inhibition of TNF-α-induced inflammatory response and LPS-induced oxidative stress by N-acetyl-d-galactosamine

**Fig. 1:**
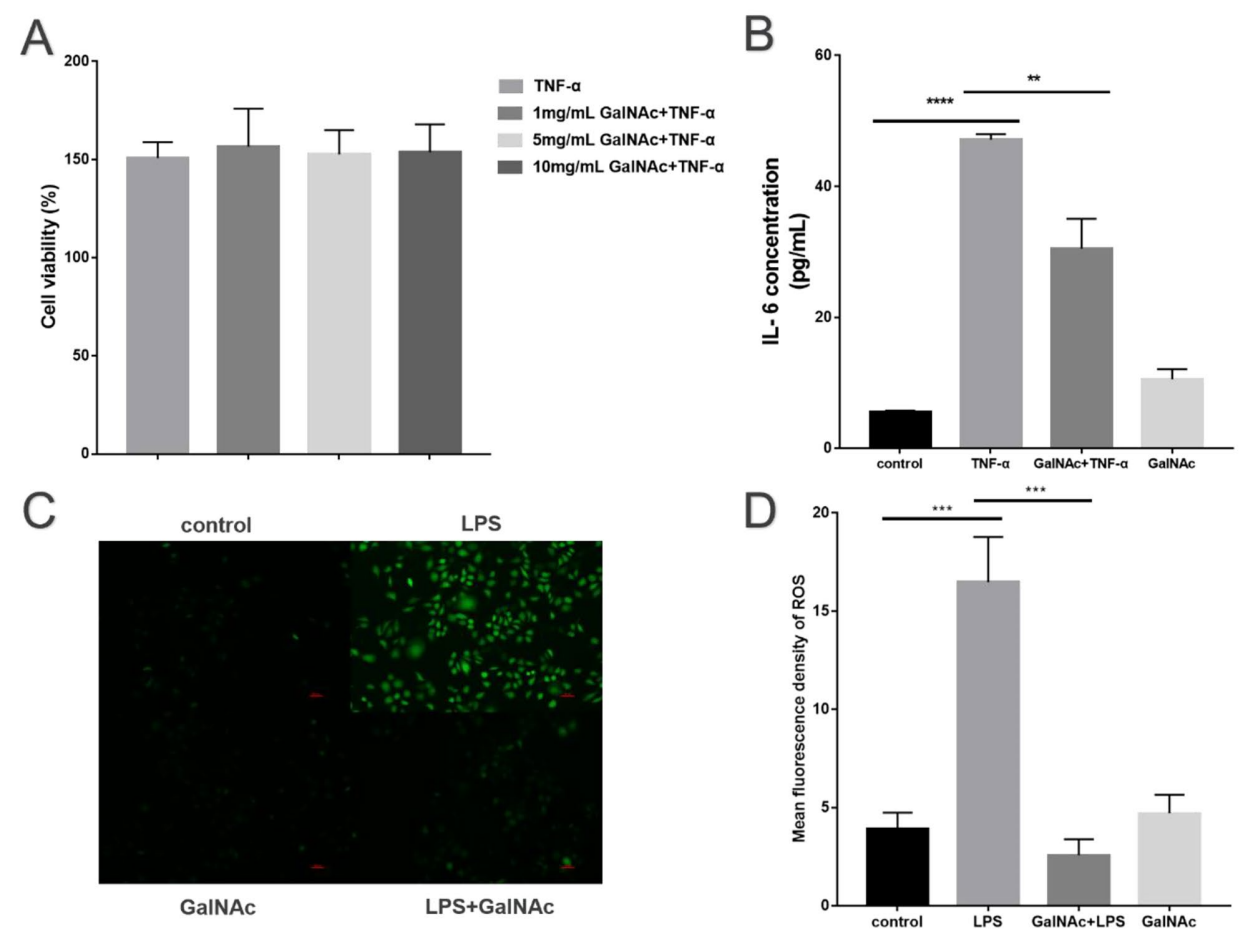
Inhibition of TNF-α-induced inflammatory response and LPS-induced oxidative stress by GalNAc. (**A**-**B**) GalNAc pretreatment reduces the expression of inflammatory factors in TNF-α-stimulated WISH cells. (**C**-**D**) GalNAc pretreatment reduces the expression of ROS in LPS-stimulated WISH cells. * < 0.05, ** < 0.01, *** < 0.001, **** < 0.0001

WISH cells were pre-treated with varying concentrations of GalNAc (1 mg/ml, 5 mg/ml, 10 mg/ml) for 2 h before exposure to TNF-α (10 ng/ml) for another 2 h. The experimental results indicated that both the TNF-α and control groups induced the proliferation of WISH cells (*P* < 0.01) in the Fig. 1A.

In the Fig. 1B, treatment of WISH cells with 10 ng/ml TNF-α significantly increased IL-6 expression levels (*P* < 0.001) compared to the control group. The expression of IL-6 induced by TNF-α was significantly decreased by GalNAc (*P* < 0.01).

The elevation of ROS in WISH cells stimulated with 1 µg/ml LPS was intervened with a high concentration of 10 mg/ml GalNAc. In the Fig. 1C, the results showed that intracellular ROS levels were significantly increased (*P* < 0.001) after LPS stimulation of WISH cells. However, after intervention with GalNAc, WISH cells showed a significant reduction in intracellular ROS levels in the LPS group in response to LPS (*P* < 0.001) in the Fig. 1D.

### GalNAc induces the synthesis of galectins outside amniotic epithelial cells

**Fig. 2:**
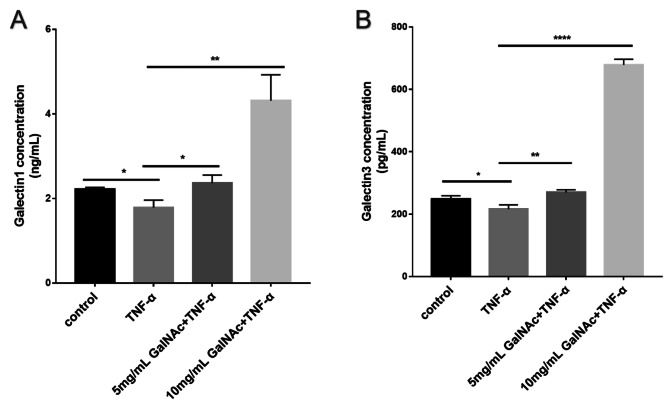
GalNAc inhibits TNF-α-stimulated inflammatory factor expression in WISH cells via galectins. (**A**) Protein level expression of Gal-1 in cell supernatant. (**B**) Protein level expression of Gal-3 in cell supernatant. * < 0.05, ** < 0.01, *** < 0.001, **** < 0.0001

As shown in Fig. 2, in the TNF-α group, the protein expression of Gal-1 (*P* < 0.05) and Gal-3 (*P* < 0.05) was significantly higher than in the control group. Furthermore, GalNAc at concentrations of 5 mg/ml and 10 mg/ml significantly increased TNF-α-induced Gal-1 (*P* < 0.05) and Gal-3 (*P* < 0.001) protein expression compared to the TNF-α group.

### GalNAc-stimulated EMT of WISH cells

**Fig. 3:**
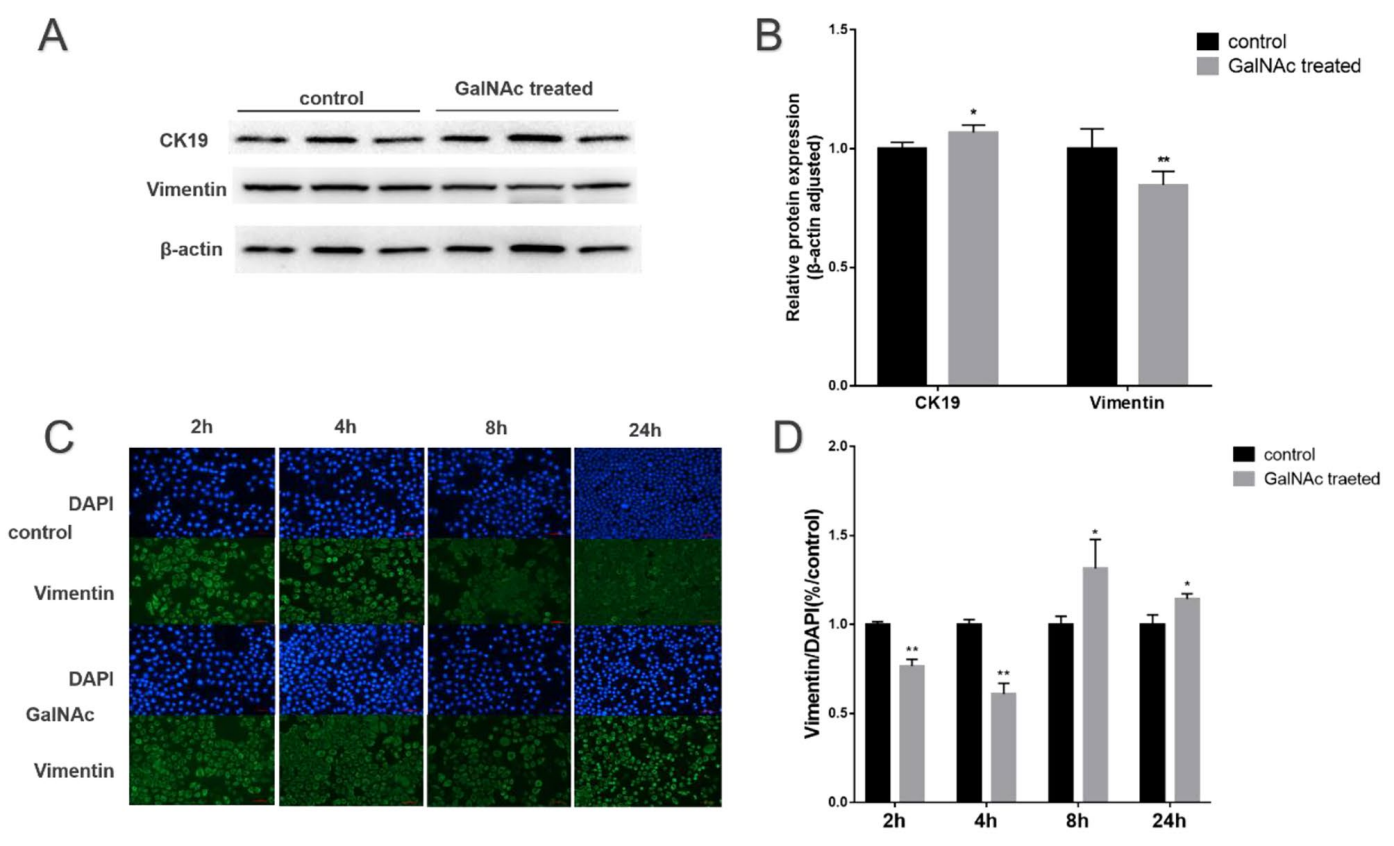
GalNAc-stimulated WISH cells EMT-MET. (**A**) Vimentin and CK-19 protein concentrations in WISH cells (from protein blotting analysis). (**B**) Vimentin and CK-19 expression analysis in WISH cells based on protein blotting results. (**C**) Immunofluorescence detection of Vimentin expression in WISH cells under 100X microscopy. (**D**) Analysis of Vimentin expression in WISH cells based on immunofluorescence results. * < 0.05, ** < 0.01, *** < 0.001, **** < 0.0001

Protein blotting experiments revealed that vimentin protein levels decreased, while CK-19 protein levels increased in the GalNAc group compared to the control group. In the Fig. 3A-B, the result suggested that the cells underwent EMT changes, but the observed trends were not statistically significant. Immunofluorescence experiments were conducted to verify vimentin expression in the cytoplasm. In the Fig. 3C-D, the results showed a trend of decreasing and then increasing within 2–24 h. Both experiments indicate that GalNAc can induce the EMT phenomenon in WISH cells.

### GalNAc did not affect the activity of hAECs and WISH cells

**Fig. 4:**
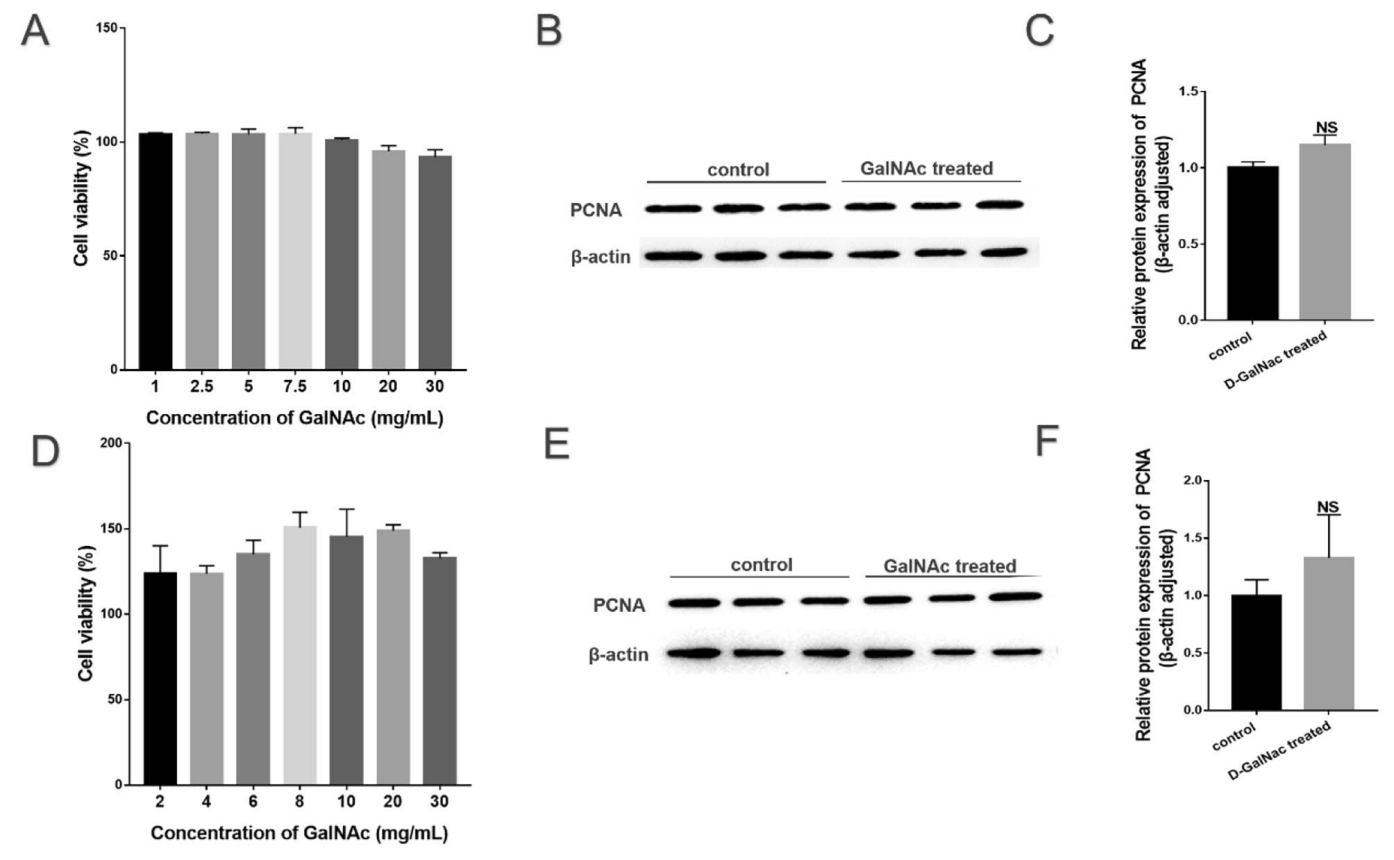
Effect of GalNAc on the activity of hAECs and WISH cells. (**A**) CCK-8 for detection of WISH cell activity. (**B**) PCNA protein concentration in WISH cells (from protein blot analysis). (**C**) PCNA expression analysis in WISH based on protein blotting results. (**D**) CCK-8 for detection of hAEC activity. (**E**) PCNA protein concentration in hAECs (from protein blot analysis). (**F**) PCNA expression analysis in hAECs based on protein blotting results. * < 0.05, ** < 0.01, *** < 0.001, **** < 0.0001

The CCK-8 assay revealed no significant difference in viability between hAECs and WISH cells in the control and GalNAc groups (*P* > 0.05, Fig. 4A and D). This indicates that GalNAc did not have a significant promotion effect on cell viability at 24 h.

The expression levels of the proliferation-associated protein PCNA in hAECs and WISH cells in the GalNAc group at 24 h were not significantly different from those in the control group (*P* > 0.05), as shown in the Fig. 4B/C and Fig. 4E/F.

### GalNAc promoted cell migration by targeting the akt pathway

**Fig. 5:**
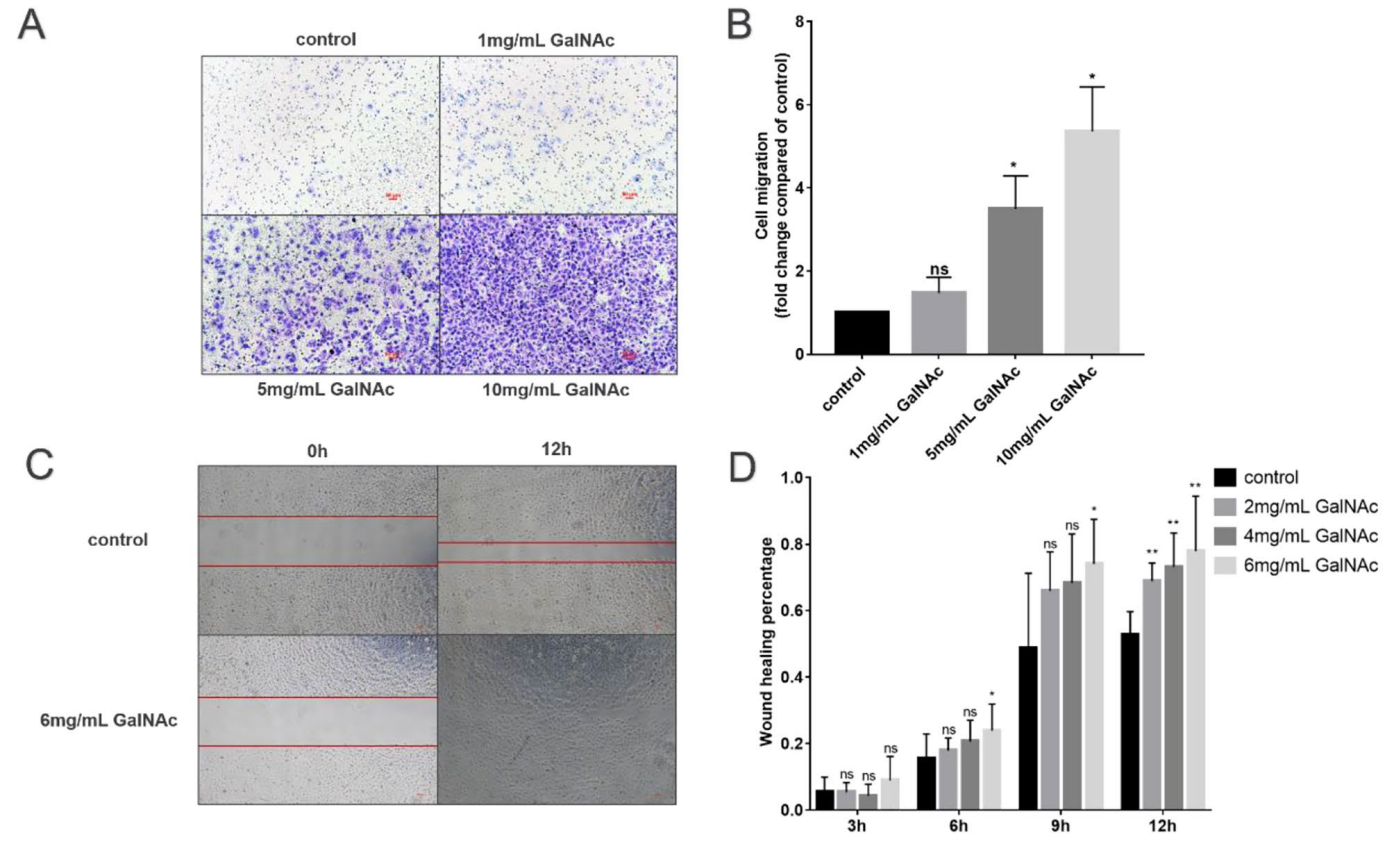
GalNAc promotes migration of hAEC and WISH cells. (**A**-**B**) Effect of GalNAc on WISH cell migration by transwell migration assay under 40X microscopy. (**C**-**D**) Detection of the effect of GalNAc on the migration of hAECs by scratch assay under 40X microscopy. * < 0.05, ** < 0.01, *** < 0.001, **** < 0.0001

As shown in the Fig. 5A-B, compared to the control group, there was no significant difference in the low to medium to high GalNAc concentration group at 3 h (*P* > 0.05). Similarly, there was no significant difference in the low to medium GalNAc group at 6 h (*P* > 0.05). However, the high GalNAc group exhibited a significant promotion of hAECs migration (*P* < 0.05). Compared to the control group, the low medium, and high GalNAc concentrations at 12 h significantly enhanced the migration of hAECs (*P* < 0.01). The closure of unrelated cells at 12 h was only 50% for the medium concentration, whereas the closure of hAECs replated with 6 mg/ml GalNAc after trauma was 80% (*P* < 0.01).

As shown in the Fig. 5C-D, the study found that low concentrations of GalNAc did not have a significant effect on the migration of WISH cells (*P* > 0.05). However, medium to high concentrations of GalNAc significantly promoted the migration of WISH cells (*P* < 0.05).

**Fig. 6:**
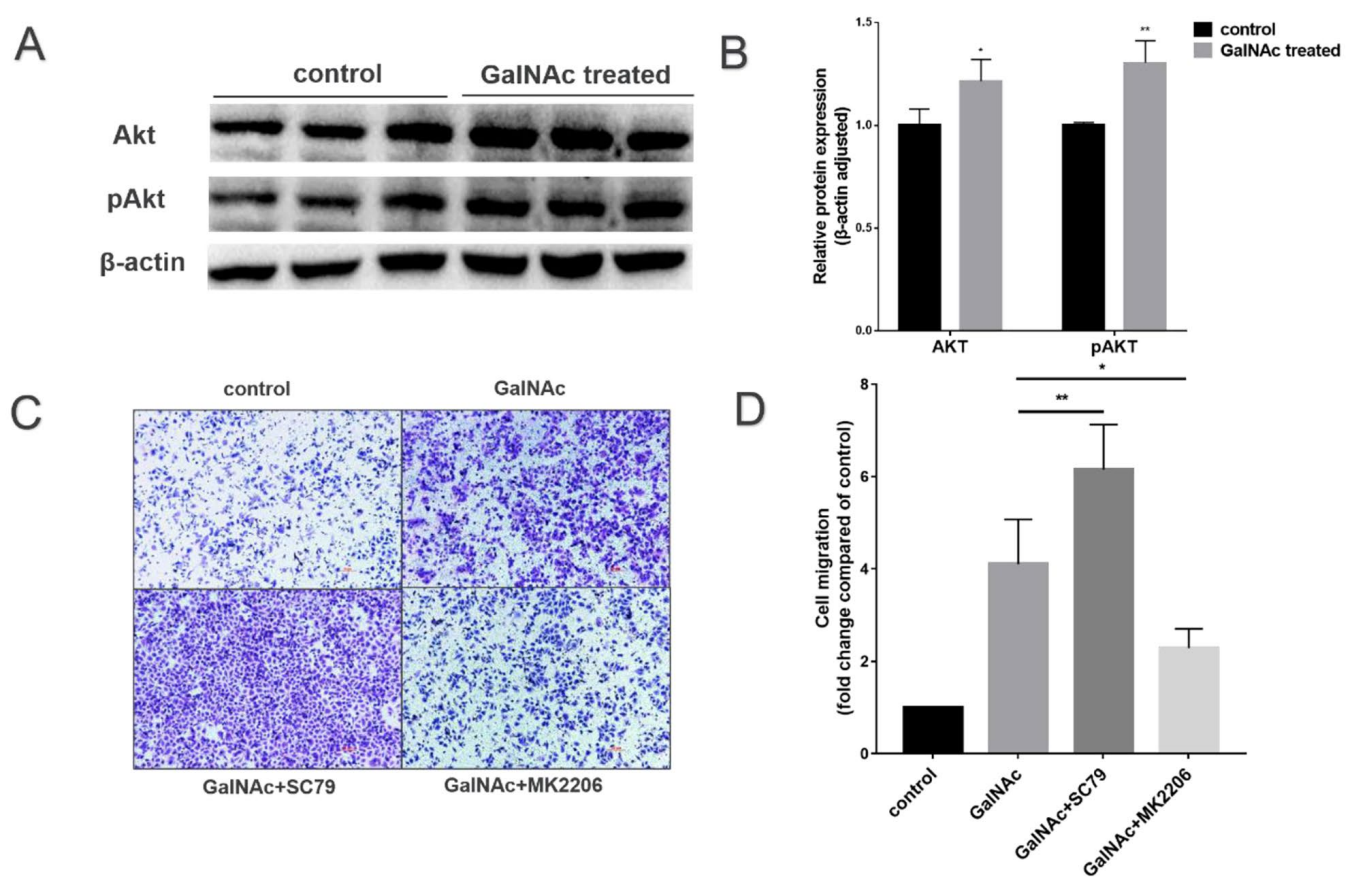
GalNAc promotes cell migration by targeting the Akt pathway. (**A**) Akt total and phosphorylated protein concentrations in WISH cells (from protein blotting analysis). (**B**) Akt and pAkt expression analysis in WISH cells based on protein blotting results. (**C**-**D**) Intervention using Akt inhibitors and activators to observe the migratory capacity of cells by transwell migration assay under 40X microscopy. *<0.05, **<0.01, ***<0.001, ****<0.0001

As shown in the Fig. 6A-B, incubation with 10 mg/ml of GalNAc was performed for 24 h. Protein blotting was used to assess the expression of Akt and pAkt. The results showed that GalNAc significantly increased the total and phosphorylated protein levels of Akt compared to the control group (*P* < 0.01). To verify whether the cells were promoting migration through the AKT signaling pathway. We conducted transwell experiments with inhibitor (MK2206) and activator (SC79) of Akt. Figure 6C-D showed that the cell migration rate decreased in the experimental group with the inhibitor added, while it increased in the experimental group with the activator added.

### GalNAc-induced sulfated glycosaminoglycan production

**Fig. 7:**
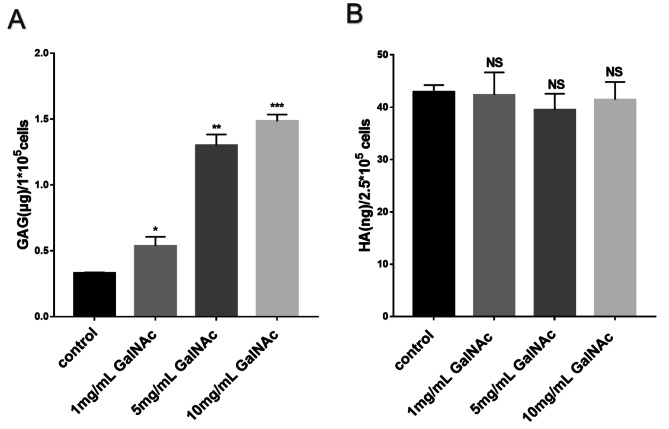
GalNAc induces sulfated glycosaminoglycan production. (**A**) GAG expression level in cell supernatant. (**B**) HA expression level in cell supernatant. * < 0.05, ** < 0.01, *** < 0.001, **** < 0.0001

Extracellular total glycosaminoglycan (GAG) levels were measured using the total GAG assay kit after administration. In the Fig. 7, the results showed that GalNAc contributed to the total GAG levels (*P*<0.05). Additionally, ELISA was used to detect the hyaluronic acid (HA) level in the corresponding extracellular supernatant. The experimental group did not show any significant changes compared to the control group (*P* > 0.05). It can be determined that GalNAc induces the production of sulfated glycosaminoglycans in the extracellular ECM of amniotic membranes. This is calculated by subtracting the HA content from the total glycosaminoglycan content.

## Discussions

In this study, we delve into the role of mammalian monosaccharide forms, such as glucose, galactose, GlcNAc, mannose, and fucose, particularly focusing on GalNAc. GalNAc is integral in various biological processes, and their significance in wound healing has been highlighted in numerous studies (Sosicka et al. 2020). For instance, GlcNAc has been shown to protect human skin fibroblasts (HS68) against UVB-induced damage and inhibit collagenase activity (Hwang et al. 2011). Similarly, local application of fucose has been found to significantly reduce MMP-9 upregulation, thereby accelerating epithelial layer recovery in iodine vapor-burnt rabbit corneas (Isnard et al. 2005). Mannose has demonstrated efficacy in reducing inflammation and neutrophil count in rat wounds (Wei et al. 2020), and galactose has been reported to enhance wound healing in rats(Kössi et al. 1999).

Monosaccharides have been employed as therapeutic alternatives for a number of wounds due to their safety, biological activities, and distinctive qualities. However, the specific role of GalNAc, a key member of the monosaccharide family, in promoting wound healing remains less explored. As an inexpensive and naturally occurring compound, GalNAc presents a promising candidate for diverse applications.

These monosaccharides, with widespread distribution in various species(Zhang et al. 2023), are found in the cytoplasm, extracellular matrix (ECM), nucleus, and on the cell surface (Camby et al. 2006). Depending on their extracellular or intracellular localization, ligand-receptor interactions reveal a wide range of biological functions, including embryogenesis, angiogenesis, cell proliferation and migration, and wound healing (Kolundžić et al. 2011; Fajka-Boja et al. 2016; Sundblad et al. 2017).Notably, a decrease in GalNAc levels has been observed in vaginal secretions of PROM patients (Liu et al. 2021a, b), along with reduced levels of Gal-1 and Gal-9 in serum(Boroń et al. 2022). The potential impact of GalNAc and GalNAc-galectins on wound healing, particularly in PROM fetal membranous tissue, has yet to be investigated.

It is well known that amniotic wound repair is consistent with other wound healing and is a systematic biological program characterized by four overlapping phases: inflammation, epithelial-mesenchymal transition (EMT), proliferation and migration (mostly migration), and remodeling, except for angiogenesis (Overmiller et al. 2022; Mogami and Word 2020). Particularly, galectins have been reported to play a role in all of these processes (Chen et al. 2022).

Notably, galectins have been implicated in each of these stages. In the inflammation stage, an excess of pro-inflammatory cytokines, proteases, and reactive oxygen species, along with pathogen-induced infections, lead to abnormal recruitment of immune cells and altered protein hydrolysis balance. The latter causes the wound to stall in the inflammatory response phase, resulting in delayed healing(Menon and Richardson 2017).The inflammation-oxidative stress axis can lead to membrane weakening through various processes (Menon and Richardson 2017), including fetal membrane aging in response to ROS, activating telomere-dependent, p38mapk signaling-driven senescence(Borodkina et al. 2014). This senescence is a mechanism that causes fetal membrane aging and results in aseptic inflammation, further damaging the membrane and potentially leading to its rupture (Menon et al. 2016).

In promoting tissue healing, the pro-inflammatory to the anti-inflammatory phase of amniotic tissue is a necessary step. In this study, it is shown that IL-6 inflammatory factor levels and ROS levels decrease after GalNAc action and that GalNAc may possess anti-inflammatory and antioxidant properties. This is similar to other previous findings that GlcNAc can reduce IL-6 levels in skin fibroblasts to heal damaged cell layers (Yang et al. 2023). Our study finds that GalNAc promotes elevated levels of extracellular Gal-1 and Gal-3. GalNAc administration increases Gal-1 and Gal-3 levels in WISH cell supernatants due to exogenous Gal-1 inhibition of LPS-stimulated release of IL-6 and IL-8 from human retinal pigment epithelial cells; and Pharmacological treatment with Gal-1 modulates acute and chronic inflammatory responses through inhibiting the migration of leukocytes and the production of pro-inflammatory cytokines, such as TNF-α, interferon-γ, IL-1β, IL-2, IL-4, IL-12 and IL-13 (Yabuta et al. 2014; La et al. 2003). Therefore, it is suggested that GalNAc exerts its anti-inflammatory and antioxidant effects in amniotic epithelial cells via extracellular Gal-1 and Gal-3, which is thought to be consistent with the anti-inflammatory and antioxidant effects of mannose-6-phosphate (Davis et al. 1994), β-glucan (El Hosary et al. 2020) and chitosan (Tiboni et al. 2022) in wound healing.

Secondly, in the epithelial-mesenchymal transition (EMT) stage, epithelial cells change their cytoskeleton and signaling pathways (Lamouille et al. 2014). The reverse process of mesenchymal-to-epithelial transition (MET) restores the specific epithelial identity (Cicchini et al. 2015). EMT and MET are well-established biological events occurring sequentially in development and organogenesis, which are reactivated and regulated in wound healing and tissue repair, in response to signals from the proximal microenvironment. Vimentin-positive cells are observed in PROM mice scattered in the epithelial layer of the mouse amniotic membrane rupture, where a process called EMT is observed at the site of rupture, in which epithelial cells acquire a mesenchymal phenotype and are associated with tissue repair (Mogami et al. 2017). In our study, we observed EMT at the site of rupture in PROM mice, where epithelial cells acquire a mesenchymal phenotype associated with tissue repair. Post-stimulation WISH cells showed an increase in CK-19 protein levels and a decrease in Vimentin, indicative of the completion of the EMT-MET process, akin to EMT changes observed in mouse skin wound healing promoted by N-acetylglucosamine(Terao et al. 2011). Furthermore, EMT mediates the migration of cultured amniotic epithelial cells through TGF-β-Smad signaling (Kawamura et al. 2022).

The third event in wound healing is the proliferation phase. The proliferation stage is characterized by epithelial proliferation and migration over the provisional matrix within the wound, which is referred to as re-epithelialization (Pizzicannella et al. 2018). Cells closest to the wound preferentially migrated, while cells far from the wound preferentially proliferated (Singer and Clark 1999). Similarly, our experiments found that GalNAc promoted wound healing in WISH cells by cell migration rather than relying on cell proliferation. Studies have shown that overexpression of Gal-1 increases phosphorylated Akt (White et al. 2014; Zhang et al. 2016) and that Akt signaling plays a critical role in both skin development and skin wound healing (Bielefeld et al. 2013). This study found that total and phosphorylated protein levels of Akt were elevated in WISH cells following GalNAc stimulation and that Akt signaling may contribute much to amniotic tissue healing. In addition, it is also demonstrated that GalNAc promotes the migratory effects of WISH cells through the Akt signaling pathway, as the effect of GalNAc on WISH cell migration can be inhibited by an Akt inhibitor (MK2206) and promoted by an Akt activator (SC79). Therefore, all these results suggest that GalNAc plays a positive role in amniotic membrane rupture healing by activating the Akt signaling pathway to promote cell migration.

Fourthly, in the remodeling stage, the termination of the wound healing process requires a fine balance between extracellular matrix (ECM) deposition and degradation (Amiri et al. 2022).PI3K/Akt pathway activation represents a key regulator of ECM production (Zhang et al. 2019). Glycosaminoglycans (GAG) are the main component of ECM (Graça et al. 2020). The sulfated glycosaminoglycan (sGAG) levels are significantly higher at non-ruptured sites of PROM fetal membranes than at ruptures (Taghiabadi et al. 2015), and GalNAc is a precursor for chondroitin sulfate and keratin sulfate biosynthesis in vivo. Therefore, this study found that exogenous GalNAc increased sGAG production but not HA production in amniotic membrane epithelial cells by 1,9-dimethyl methylene blue (DMMB) colorimetric assay and ELISA, which is similar to the result that exogenous GlcNAc increased hyaluronic acid production in human dermal fibroblasts (Tu et al. 2009). Since Gal-3 is featured by a glycosaminoglycan binding protein (GAGBP), it can interact with sGAG, and chondroitin sulfate proteoglycan (CSPG) (Edwards et al. 2022). Thus, GalNAc not only alters ECM components but also promotes amniotic tissue remodeling through the galectin pathway. Activation of the PI3K/Akt pathway is a key regulator of ECM production.

## Conclusions

The role of GalNAc stimulation of extracellular galectin production in the regulation of the physiological process of amniotic membrane wound healing is described. In the results, it is found that GalNAc enhances extracellular galectins production, which triggers different phases of amniotic membrane tissue repair, including inflammation (inhibition of cellular inflammation and oxidative stress); proliferation, migration re-epithelialization (promotion of cellular EMT and migration via the Akt signaling pathway); and regression, remodeling, and healing (synthesis of sGAG, alteration of ECM composition). This study indicates that galectins may be crucial in the promotion of GalNAc’s ability to stimulate amniotic tissue healing.

## Data Availability

Not applicable.

## References

[CR47] Amiri N, Golin AP, Jalili RB, Ghahary A. Roles of cutaneous cell-cell communication in wound healing outcome: an emphasis on keratinocyte-fibroblast crosstalk. Exp Dermatol. 2022;31(4):475–84. 34932841 10.1111/exd.14516

[CR46] Bielefeld KA, Amini-Nik S, Alman BA. Cutaneous wound healing: recruiting developmental pathways for regeneration, Cellular and molecular life sciences. CMLS. 2013;70(12):2059–81. 23052205 10.1007/s00018-012-1152-9 PMC3663196

[CR29] Borodkina A, Shatrova A, Abushik P, Nikolsky N, Burova E. Interaction between ROS dependent DNA damage, mitochondria and p38 MAPK underlies senescence of human adult stem cells. Aging. 2014;6(6):481–95. 24934860 10.18632/aging.100673 PMC4100810

[CR26] Boroń DG, Świetlicki A, Potograbski M, Kurzawińska G, Wirstlein P, Boroń D, Drews K, Seremak-Mrozikiewicz A. Galectin-1 and Galectin-9 concentration in maternal serum: implications in pregnancies complicated with Preterm Prelabor rupture of membranes. J Clin Med 11(21) (2022). 10.3390/jcm11216330 PMC9658671 36362558

[CR21] Camby I, Le Mercier M, Lefranc F, Kiss R. Galectin-1: a small protein with major functions. Glycobiology. 2006;16(11):r137–57. 10.1093/glycob/cwl025 16840800

[CR27] Chen JL, Chen Y, Xu DX, Chen DZ. Possible important roles of galectins in the healing of human fetal membranes. Front Endocrinol (Lausanne). 2022;13:941029. 36017312 10.3389/fendo.2022.941029 PMC9395672

[CR38] Cicchini C, Amicone L, Alonzi T, Marchetti A, Mancone C, Tripodi M. Molecular mechanisms controlling the phenotype and the EMT/MET dynamics of hepatocyte. Liver International: Official J Int Association Study Liver. 2015;35(2):302–10. 10.1111/liv.12577 PMC4344819 24766136

[CR34] Davis RH, Donato JJ, Hartman GM, Haas RC. Anti-inflammatory and wound healing activity of a growth substance in Aloe vera. J Am Podiatr Med Assoc. 1994;84(2):77–81. 8169808 10.7547/87507315-84-2-77

[CR12] Dawson JS. Preiskel Elective Prize. The role of sugar in wound healing. A comparative trial of the healing of infected wounds using traditional gauze/antiseptic packing, and granulated sugar. Undertaken Dur Elect Period Kagando Hosp Uganda Annals Royal Coll Surg Engl. 1996;78(2 Suppl):82–5. 8687073

[CR52] Edwards JL, Kadav PD, Bandyopadhyay P, Dam TK. Revealing the identity of human Galectin-3 as a glycosaminoglycan-binding protein. (Clifton N J). 2022;2442:137–50. Methods in molecular biology. 10.1007/978-1-0716-2055-7_8 35320524

[CR35] El Hosary R, El-Mancy SMS, El Deeb KS, Eid HH, El Tantawy ME, Shams MM, Samir R, Assar NH, Sleem AA. Efficient wound healing composite hydrogel using Egyptian Avena sativa L. polysaccharide containing β-glucan. Int J Biol Macromol. 2020;149:1331–8. 31712156 10.1016/j.ijbiomac.2019.11.046

[CR23] Fajka-Boja R, Urbán VS, Szebeni GJ, Czibula Á, Blaskó A, Kriston-Pál É, Makra I, Hornung Á, Szabó E, Uher F, Than NG, Monostori É. Galectin-1 is a local but not systemic immunomodulatory factor in mesenchymal stromal cells. Cytotherapy. 2016;18(3):360–70. 26857229 10.1016/j.jcyt.2015.12.004

[CR10] Forrest RD. Early history of wound treatment. J R Soc Med. 1982;75(3):198–205. 7040656 10.1177/014107688207500310 PMC1437561

[CR49] Graça MFP, Miguel SP, Cabral CSD, Correia IJ. Hyaluronic acid-based wound dressings: a review. Carbohydr Polym. 2020;241:116364. 32507198 10.1016/j.carbpol.2020.116364

[CR16] Hwang YP, Kim HG, Han EH, Choi JH, Park BH, Jung KH, Shin YC, Jeong HG. N-Acetylglucosamine suppress collagenases activation in ultraviolet B-irradiated human dermal fibroblasts: involvement of calcium ions and mitogen-activated protein kinases. J Dermatol Sci. 2011;63(2):93–103. 21600739 10.1016/j.jdermsci.2011.04.008

[CR17] Isnard N, Bourles-Dagonet F, Robert L, Renard G. Studies on corneal wound healing. Effect of fucose on iodine vapor-burnt rabbit corneas, Ophthalmologica. Journal international d’ophtalmologie. International journal of ophthalmology. Z fur Augenheilkunde. 2005;219(6):324–33. 10.1159/000088373 16286790

[CR3] Johnson JW, Egerman RS, Moorhead J. Cases with ruptured membranes that reseal, Am J Obstet Gynecol 163(3) (1990) 1024-30; discussion 1030-2. 10.1016/0002-9378(90)91117-u 2206055

[CR41] Kawamura Y, Mogami H, Yasuda E, Takakura M, Matsuzaka Y, Ueda Y, Inohaya A, Kawasaki K, Chigusa Y, Mandai M, Kondoh E. Fetal macrophages assist in the repair of ruptured amnion through the induction of epithelial-mesenchymal transition. Sci Signal. 2022;15(751):eabi5453. 36099339 10.1126/scisignal.abi5453

[CR22] Kolundžić N, Bojić-Trbojević Z, Radojčić L, Petronijević M, Vićovac L. Galectin-8 is expressed by villous and extravillous trophoblast of the human placenta. Placenta. 2011;32(11):909–11. 21862124 10.1016/j.placenta.2011.07.087

[CR19] Kössi J, Peltonen J, Ekfors T, Niinikoski J, Laato M. Effects of hexose sugars: glucose, fructose, galactose and mannose on wound healing in the rat, European surgical research. Europaische Chirurgische Forschung Recherches Chirurgicales Europeennes. 1999;31(1):74–82. 10072613 10.1159/000008623

[CR33] La M, Cao TV, Cerchiaro G, Chilton K, Hirabayashi J, Kasai K, Oliani SM, Chernajovsky Y, Perretti M. A novel biological activity for galectin-1: inhibition of leukocyte-endothelial cell interactions in experimental inflammation. Am J Pathol. 2003;163(4):1505–15. 14507657 10.1016/S0002-9440(10)63507-9 PMC1868297

[CR37] Lamouille S, Xu J, Derynck R. Molecular mechanisms of epithelial-mesenchymal transition, Nature reviews. Mol cell Biology. 2014;15(3):178–96. 10.1038/nrm3758 PMC4240281 24556840

[CR14] Liu L, Xu HJ, Chen JL, Chen Z, Zhan HY, Xu DX, Chen Y, Xu ZF, Chen DZ. Detection of vaginal metabolite changes in premature rupture of membrane patients in third trimester pregnancy: a prospective Cohort Study, Reproductive sciences (Thousand Oaks. Calif). 2021a;28(2):585–94. 10.1007/s43032-020-00338-9 PMC7537967 33025530

[CR25] Liu L, Chen Y, Chen JL, Xu HJ, Zhan HY, Chen Z, Chen DZ, Xu ZF, Xu DX. Integrated metagenomics and metabolomics analysis of third-trimester pregnant women with premature membrane rupture: a pilot study. Annals Translational Med. 2021b;9(23):1724. 10.21037/atm-21-5539 PMC8743720 35071418

[CR28] Menon R, Richardson LS. Preterm prelabor rupture of the membranes: a disease of the fetal membranes. Semin Perinatol. 2017;41(7):409–19. 28807394 10.1053/j.semperi.2017.07.012 PMC5659934

[CR30] Menon R, Behnia F, Polettini J, Saade GR, Campisi J, Velarde M. Placental membrane aging and HMGB1 signaling associated with human parturition. Aging. 2016;8(2):216–30. 26851389 10.18632/aging.100891 PMC4789578

[CR13] Migone C, Scacciati N, Grassiri B, De Leo M, Braca A, Puppi D, Zambito Y, Piras AM. Jellyfish Polysaccharides for Wound Healing Applications. Int J Mol Sci 23(19) (2022). 10.3390/ijms231911491 PMC9569628 36232791

[CR7] Mogami H, Word RA. Healing mechanism of ruptured fetal membrane. Front Physiol. 2020;11:623. 32625113 10.3389/fphys.2020.00623 PMC7311775

[CR39] Mogami H, Hari Kishore A, Akgul Y, Word RA. Healing Preterm Ruptured Fetal Membr Sci Rep. 2017;7(1):13139. 10.1038/s41598-017-13296-1 PMC5640674 29030612

[CR5] Overmiller AM, Sawaya AP, Hope ED, Morasso MI. Intrinsic Networks Regulating Tissue Repair: Comparative Studies of Oral and Skin Wound Healing, Cold Spring Harbor perspectives in biology 14(11) (2022). 10.1101/cshperspect.a041244 PMC9620853 36041785

[CR4] Parry S, Strauss JF 3. Premature rupture of the fetal membranes. N Engl J Med. 1998;338(10):663–70. 9486996 10.1056/NEJM199803053381006

[CR6] Pasquier JC, Doret M. Fetal membranes: embryological development, structure and the physiopathology of the preterm premature rupture of membranes. J Gynecol Obstet Biol Reprod. 2008;37(6):579–88. 10.1016/j.jgyn.2007.12.001 18424017

[CR11] Pieper B, Caliri MH. Nontraditional wound care: a review of the evidence for the use of sugar, papaya/papain, and fatty acids, Journal of wound, ostomy, and continence nursing: official publication of the Wound. Ostomy Cont Nurses Soc. 2003;30(4):175–83. 10.1067/mjw.2003.131 12851592

[CR42] Pizzicannella J, Diomede F, Merciaro I, Caputi S, Tartaro A, Guarnieri S, Trubiani O. Endothelial committed oral stem cells as modelling in the relationship between periodontal and cardiovascular disease. J Cell Physiol. 2018;233(10):6734–47. 29600566 10.1002/jcp.26515

[CR9] Quintero RA, Carreño CA, Yelian F, Evans MI. Repair kinetics of amnion cells after microsurgical injury. Fetal Diagn Ther. 1996;11(5):348–56. 8894631 10.1159/000264340

[CR2] Ronzoni S, Cobo T, D’Souza R, Asztalos E, O’Rinn SE, Cao X, Herranz A, Melamed N, Ferrero S, Barrett J, Aldecoa V, Palacio M. Individualized treatment of preterm premature rupture of membranes to prolong the latency period, reduce the rate of preterm birth, and improve neonatal outcomes. Am J Obstet Gynecol. 2022;227(2):e2961–29618. 10.1016/j.ajog.2022.02.037 35257664

[CR43] Singer AJ, Clark RA. Cutaneous wound healing. N Engl J Med. 1999;341(10):738–46. 10471461 10.1056/NEJM199909023411006

[CR8] Sonnemann KJ, Bement WM. Wound repair: toward understanding and integration of single-cell and multicellular wound responses. Annu Rev Cell Dev Biol. 2011;27:237–63. 21721944 10.1146/annurev-cellbio-092910-154251 PMC4878020

[CR15] Sosicka P, Ng BG, Freeze HH. Therapeutic monosaccharides: looking back, moving Forward. Biochemistry. 2020;59(34):3064–77. 31398011 10.1021/acs.biochem.9b00565 PMC7282196

[CR24] Sundblad V, Morosi LG, Geffner JR, Rabinovich GA. Galectin-1: a Jack-of-All-trades in the resolution of Acute and chronic inflammation. J Immunol (Baltimore Md : 1950). 2017;199(11):3721–30. 10.4049/jimmunol.1701172 29158348

[CR50] Taghiabadi E, Nasri S, Shafieyan S, Jalili Firoozinezhad S, Aghdami N. Fabrication and characterization of spongy denuded amniotic membrane based scaffold for tissue engineering. Cell J. 2015;16(4):476–87. 25685738 10.22074/cellj.2015.493 PMC4297486

[CR1] Tchirikov M, Schlabritz-Loutsevitch N, Maher J, Buchmann J, Naberezhnev Y, Winarno AS, Seliger G. Mid-trimester preterm premature rupture of membranes (PPROM): etiology, diagnosis, classification, international recommendations of treatment options and outcome. J Perinat Med. 2018;46(5):465–88. 28710882 10.1515/jpm-2017-0027

[CR40] Terao M, Ishikawa A, Nakahara S, Kimura A, Kato A, Moriwaki K, Kamada Y, Murota H, Taniguchi N, Katayama I, Miyoshi E. Enhanced epithelial-mesenchymal transition-like phenotype in N-acetylglucosaminyltransferase V transgenic mouse skin promotes wound healing. J Biol Chem. 2011;286(32):28303–11. 21697088 10.1074/jbc.M111.220376 PMC3151074

[CR36] Tiboni M, Elmowafy E, El-Derany MO, Benedetti S, Campana R, Verboni M, Potenza L, Palma F, Citterio B, Sisti M, Duranti A, Lucarini S, Soliman ME, Casettari L. A combination of sugar esters and chitosan to promote in vivo wound care. Int J Pharm. 2022;616:121508. 35123002 10.1016/j.ijpharm.2022.121508

[CR51] Tu CX, Zhang RX, Zhang XJ, Huang T. Exogenous N-acetylglucosamine increases hyaluronan production in cultured human dermal fibroblasts. Arch Dermatol Res. 2009;301(7):549–51. 19247681 10.1007/s00403-009-0932-z

[CR18] Wei Z, Huang L, Cui L, Zhu X. Mannose: good player and assister in pharmacotherapy. Biomed Pharmacotherapy = Biomedecine Pharmacotherapie. 2020;129:110420. 32563989 10.1016/j.biopha.2020.110420

[CR44] White NM, Masui O, Newsted D, Scorilas A, Romaschin AD, Bjarnason GA, Siu KW, Yousef GM. Galectin-1 has potential prognostic significance and is implicated in clear cell renal cell carcinoma progression through the HIF/mTOR signaling axis. Br J Cancer. 2014;110(5):1250–9. 24496460 10.1038/bjc.2013.828 PMC3950857

[CR32] Yabuta C, Yano F, Fujii A, Shearer TR, Azuma M. Galectin-3 enhances epithelial cell adhesion and wound healing in rat cornea. Ophthalmic Res. 2014;51(2):96–103. 24356704 10.1159/000355846

[CR31] Yang H, Yu X, Liu J, Tao Y, Nong G. Investigation of the structure of gallate xylose polymers and their antioxidant properties for skin care products. Carbohydr Res. 2023;523:108728. 36473322 10.1016/j.carres.2022.108728

[CR45] Zhang PF, Li KS, Shen YH, Gao PT, Dong ZR, Cai JB, Zhang C, Huang XY, Tian MX, Hu ZQ, Gao DM, Fan J, Ke AW, Shi GM. Galectin-1 induces hepatocellular carcinoma EMT and sorafenib resistance by activating FAK/PI3K/AKT signaling. Cell Death Dis. 2016;7(4):e2201. 27100895 10.1038/cddis.2015.324 PMC4855644

[CR48] Zhang J, Zhou Q, Wang H, Huang M, Shi J, Han F, Cai W, Li Y, He T, Hu D. MicroRNA-130a has pro-fibroproliferative potential in hypertrophic scar by targeting CYLD. Arch Biochem Biophys. 2019;671:152–61. 31283910 10.1016/j.abb.2019.07.003

[CR20] Zhang C, Liu H, Sun L, Wang Y, Chen X, Du J, Sjöling Å, Yao J, Wu S. An overview of host-derived molecules that interact with gut microbiota, iMeta (2023). 10.1002/imt2.88 PMC10989792 38868433

